# Poisoning with Thyroid Hormones Used Illegally—Systematic Review

**DOI:** 10.3390/ph18121808

**Published:** 2025-11-27

**Authors:** Monika Skrzypiec-Spring, Krzysztof Kujawa, Anna Wietrzyk, Paulina Matuła, Magdalena Materna, Wiktoria Michalska, Dorota Szumny, Adam Szeląg

**Affiliations:** 1 Department of Pharmacology, Faculty of Medicine, Wroclaw Medical University, 50-345 Wroclaw, Poland; anna.wietrzyk@student.umw.edu.pl (A.W.); paulina.matula@student.umw.edu.pl (P.M.); magdalena.materna@student.umw.edu.pl (M.M.); wiktoria.michalska@student.umw.edu.pl (W.M.); dorota.szumny@umw.edu.pl (D.S.); adam.szelag@umw.edu.pl (A.S.); 2 Statistical Analysis Centre, Wroclaw Medical University, 50-367 Wroclaw, Poland; krzysztof.kujawa@umw.edu.pl

**Keywords:** thyroid hormones, drug abuse, poisoning, toxicological effects, public health impact

## Abstract

**Background/Objectives**: Thyroid hormones, considered safe in therapeutic doses, are used to treat hypothyroidism, a common condition. Due to a combination of factors, including their mechanism of action, availability, and low price, these drugs are used illegally, mainly to improve performance, to assist in weight loss, or for attempting suicide. Their overuse can lead to serious health consequences, including death. Although thyroid hormones are abused, there are no studies assessing the scale, characteristics, and consequences of their illegal use. The aim of this study was to evaluate case reports of thyroid hormone poisoning from the last 30 years, assessing their dynamics and characteristics. **Methods**: Full-text clinical case studies were obtained by searching PubMed, Google Scholar, MEDLINE, Embase, Web of Science, and Scopus for the following terms: “thyroid hormones”, “thyroxine”, “levothyroxine”, “triiodothyronine”, and “liothyronine”, as well as “intoxication”, “overdose”, and “poisoning”. This study adhered to Preferred Reporting for Systematic Reviews and Meta-analyses (PRISMA) guidelines for systematic reviews. **Results**: Thyroid hormones are abused particularly by athletes, persons trying to lose weight, or those attempting suicide. There has been an upward trend in thyroid hormone poisoning over the past 30 years, particularly since 2015. The same trend has been observed in cases of thyroid hormone use for doping, among other performance-enhancing drugs. Thyroid hormone use for doping was the most common cause of poisoning with these drugs, with other clinical manifestations from poisonings due to other causes. No upward trend has been observed in the use of thyroid hormones in suicide attempts since 2017, as this number remains stable. **Conclusions**: Although exploratory in nature, our work indicates that thyroid hormone poisoning, associated mostly with the illegal use of anabolic–androgenic steroids, exhibits an increasing tendency. Moreover, thyroid hormone abuse is an important issue in suicidology.

## 1. Introduction

The changes in the world caused by the digital revolution and advances in medicine and human knowledge have both positive and negative consequences. The media create idealized images of men and women. Striving to meet unrealistic standards can lead to serious mental health problems, including eating disorders and depression, with the most serious consequences being suicide attempts and excessive physical activity [1,2]. The development of medicine and pharmacology entails people often turning to these fields of science for ideal appearance and well-being. In sports, this leads to the use of illegal pharmacological substances aimed at enhancing athletic performance, leading to addiction and poisoning. A particularly interesting group of drugs whose importance in addiction medicine, toxicology, and sports medicine has not yet been sufficiently researched are thyroid hormones.

Thyroid hormones exert regulatory influence on many physiological systems across various tissues. They have a permissive effect on catecholamines by increasing the expression of beta receptors, which leads to elevated heart rate, stroke volume, and cardiac contractility [3]. They stimulate mitochondrial function, causing increased cellular oxidative capacity, and stimulate mitochondrial oxidative phosphorylation, increasing ATP production [4]. They regulate enzymes involved in lipolysis, lipogenesis, gluconeogenesis, glycogenolysis, and insulin-dependent glucose uptake, increasing the supply of energy substrates during prolonged physical exercise [5,6]. Moreover, they regulate the growth and differentiation of fast-twitch type II muscle fibers, which are essential for performing powerful movements [6]. Additionally, thyroid hormones enhance the speed of skeletal muscle contraction and relaxation, influence the energy efficiency of contraction through greater ATP consumption at rest and during activity, and improve/elevate glycolytic capacity and mitochondrial density, intensifying ATP generation [7]. They also help accelerate recovery and reduce post-exercise muscle discomfort by stimulating protein synthesis and improving the turnover of damaged proteins [8]. In adipocytes, triiodothyronine influences lipid turnover and appetite regulation. Preclinical studies suggest that thyromimetics may be useful in the treatment of obesity and dyslipidemia [9].

All these effects of thyroid hormones may explain their appeal for bodybuilders, who expect their beneficial effects on reducing body fat and muscle mass, as well as in individuals wishing to lose weight. Although thyroid hormones are not officially approved for the treatment of obesity, they remain appealing to those aiming for weight management and increased calorie burn, such as athletes in sports that require low body fat [10,11]. This can lead to factitious thyrotoxicosis, a common form of thyroid hormone abuse, which occurs when it is taken voluntarily but covertly in excessive doses to reduce body fat and weight while maintaining apparent physical fitness. It can also occur in Munchausen syndrome, where the goal is to draw attention to the need for treatment. Thyroid hormone abuse also occurs among competitive athletes and bodybuilders who want to achieve a desired weight and low body fat. In turn, thyroid dysfunction can affect cognitive abilities and mental health, and there are studies confirming the link between thyroid disease and suicide attempts [12]. Their use for suicidal purposes may stem from the fact that thyroid hormones play an important role in regulating mood, and disorders of these hormones, especially hypothyroidism, can lead to depressive symptoms.

For weight loss purposes, thyroid hormones are sometimes used by doctors in patients with normal thyroid function, even though this is inconsistent with applicable standards. A survey conducted among thyroid specialists who were members of national endocrinology and/or thyroid research organizations from European countries with populations of over four million showed that approximately 5% of respondents believed that thyroid hormones may be indicated for the treatment of obesity in euthyroid patients [11].

Abusing thyroid hormones to lose weight is not common practice, but it does occur. The scale of thyroid hormone use for weight loss is unknown because, to the authors’ knowledge, there are no population studies that have assessed this problem. Similarly, there are no studies assessing how often these drugs are used in suicide attempts. The complex issue of using thyroid hormones in sports is also poorly understood. So far, according to World Anti-Doping Agency (WADA), thyroid hormones are not considered to be doping agents. Based on the current regulations, according to Gilda et al., thyroid hormones do not meet two of the three criteria required by the WADA Code for inclusion on the Prohibited List: (1) having the potential to enhance athletic performance, (2) posing an actual or potential health risk, and (3) violating the spirit of sport [13]. This verdict has been caused by the lack of convincing evidence regarding the prevalence of thyroid hormone use among competitive athletes, and especially the questions of whether the hormone may affect athletic performance [14]. To the authors’ knowledge, only two studies have attempted to assess the scale of thyroid hormone use in professional sports. Based on the analysis of thyroid hormone concentrations, Handelsman et al. found out that thyrotoxicosis occurred in 4/1000 athletes [15]. They also discovered that 4/1000 athletes reported using levothyroxine (T4). Due to data protection issues, it was not possible to determine whether thyrotoxicosis occurred in individuals who reported using T4. In another study, based on the analysis of data from the Doping Control Form, the prevalence of thyroid hormone use among athletes who participated in the Pyeongchang 2018 Winter Olympics, the Minsk 2019 European Games, the Tokyo 2020 Olympics, and the Paralympic Games was 1.4%, slightly higher than the prevalence of thyroid hormone use in the general community [16]. It is obvious that the development of the Internet has made protocols regarding the illegal use of thyroid hormones widely available on websites aimed at bodybuilders and athletes participating in weight-class sports [17]. Even in countries with strict regulations regarding drug distribution, there is unfortunately unrestricted access to dietary supplements on websites [17]. In spite of this, to the authors’ knowledge, in amateur sports, there have been no studies conducted to assess the scale and nature of illegal use of thyroid hormones. Information on this topic can only be found in studies examining the illegal use of anabolic–androgenic steroids, where thyroid hormones may be one of the elements of complex “steroid cycles”. In our previous study, we showed that 35.42% of people practicing amateur strength sports or training at municipal gyms who illegally used testosterone also used thyroid hormones [18]. Unfortunately, we did not specify the characteristics of thyroid hormone use in that study.

Although thyroid hormones are abused, there are no studies assessing the scale, characteristics, and consequences of their illegal use. The aim of this study was to systematically review clinical case reports of thyroid hormone poisoning from the last 30 years, evaluating their dynamics, causes, and characteristics.

## 2. Materials and Methods

The study adhered to Preferred Reporting for Systematic Reviews and Meta-analyses 2020 (PRISMA 2020) guidelines for systematic reviews (Supplementary Table S1) [19].

### 2.1. Search Strategy

The literature on thyroid hormone poisoning was retrieved from databases: PubMed, Google Scholar, MEDLINE, Embase, Web of Science, and Scopus. The search strategy consisted of using the keywords “thyroid hormones”, “thyroxine”, “levothyroxine”, “triiodothyronine”, and “liothyronine”, as well as “intoxication”, “overdose”, and “poisoning”. The search string, the same for all the databases, was developed as follows: (intoxication OR overdose OR poisoning) AND (thyroid hormones OR thyroxine OR levothyroxine OR triiodothyronine OR liothyronine). The search was restricted to studies published between the years 1995 and 2025. The last search was conducted on 30 October 2025. To avoid duplication, specific Excel columns containing digital object identifiers (DOIs) were checked for duplicates, and then duplicate rows were removed, leaving only the first occurrence of each unique record.

### 2.2. Selection Criteria

The inclusion criteria for the studies were as follows:

- Type of study: full-text clinical case studies, defined as observational studies that analyze one to four clinical cases, providing a detailed description of patient demographics, relevant clinical data, and symptoms in order to provide information on rare or unknown diseases, clinical situations, and complications.
- Clinical data required: age, gender, type of thyroid hormone used, dosage method, purpose of the use of thyroid hormones, and symptoms.
- Population: adults.
- Language: English.

The exclusion criteria were

- Publication type other than a clinical case study as defined above, with no access to the full-text version of the article;
- Lack of required clinical data listed above;
- Study population of children and adolescents;
- Publication in a language other than English.

The selection of studies was carried out by 2 teams of 2 researchers, based on the inclusion and exclusion criteria. Then, selected full-text articles were subjected to second-stage screening. Full-text screening was performed, and the selected studies were then submitted for quality check.

### 2.3. Quality Assessment

The quality of the selected articles was evaluated with the use of the Joanna Briggs Institute (JBI) critical appraisal checklists. Each option was scored as 1 point for “Yes” and 0 points for “No/Unclear/Not Applicable.” The scores were then tallied and converted into percentages. Each article could receive a quality score ranging from 10% to 100%. Articles with a score of 80.00–100% were regarded as good articles, those between 50.00% and 80.00% as sufficient articles, and those <50.00% as poor articles. All the articles selected for data extraction were rated as good; therefore, it was not necessary to create subgroups for statistical analysis.

### 2.4. Data Extraction

A data extraction format was prepared. The items included were DOI number, study title, year of publication, the country of origin, patient age, patient gender, type of thyroid hormone used, dosage method, doses used, purpose of thyroid hormones’ use, symptoms, pre-existing thyroid disease, prior treatment with thyroid hormones, co-morbidities, concomitant use of other drugs, and outcome. Each clinical case from the multi-patient cases was considered separately.

The data were independently extracted by 2 teams of 2 researchers and were compared for their integrity and correctness.

**Figure 1:**
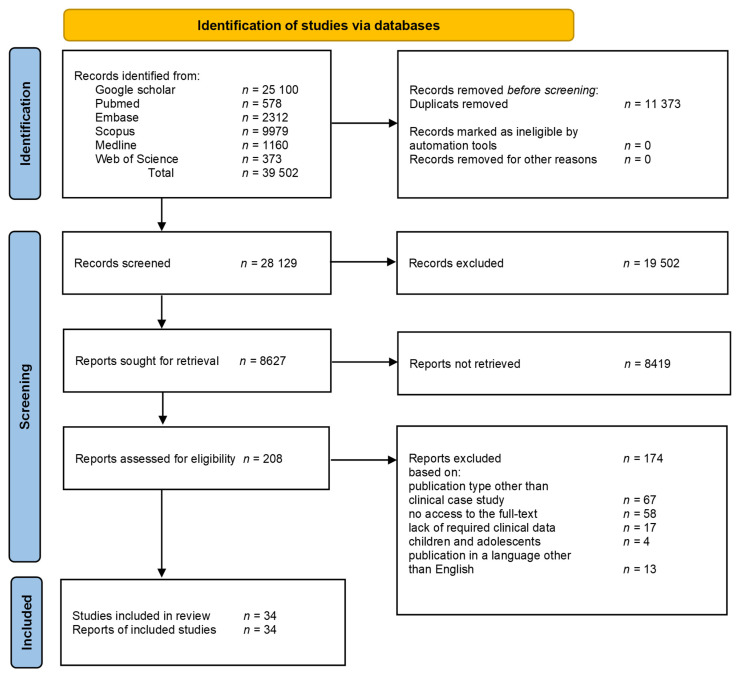
Flowchart of the search strategy.

The process of search has been depicted with the use of the PRISMA flowchart as shown in Figure 1.

As the search covered only half of the year 2025, the analysis of the trend in the number of cases per year was performed excluding the data from 2025. The other analyses were conducted using the whole dataset.

The analysis of trends in the number of cases published per year was carried out with the use of Spearman correlation (package “stats”). The relationships between categorical variables were analyzed with the use of Fisher’s exact test (“stats”). The difference in age between the group which used thyroid hormones for doping and the group which used these hormones for another reason was tested with the Wilcoxon rank sum test (“stat”), and the difference in the variance between these groups with the Levene test (“car The Fisher’s exact test does not demand any assumptions”). As the data distribution in the compared groups deviated from the normal one, the Levene test was used with the option of the median value as the central tendency measure. Fisher’s exact test of independence of relationships between categorial variables should be considered exploratory analysis; therefore, no correction of the *p*-values was used. As the reason for using thyroid hormones was both doping and suicide in one case, the case was excluded from the analyses based on distinguishing the reason for using these hormones (doping/other). The statistical analysis was performed using R 4.4.2.

## 3. Results

In the analyzed period from 1995 to 2025, 34 clinical cases of thyroid hormone poisoning were reported (Supplementary Table S2) [20,21,22,23,24,25,26,27,28,29,30,31,32,33,34,35,36,37,38,39,40,41,42,43,44,45,46,47,48,49].

### 3.1. Temporal Fluctuations in the Number of Clinical Cases of Thyroid Hormone Poisoning over the Last 30 Years

**Figure 2:**
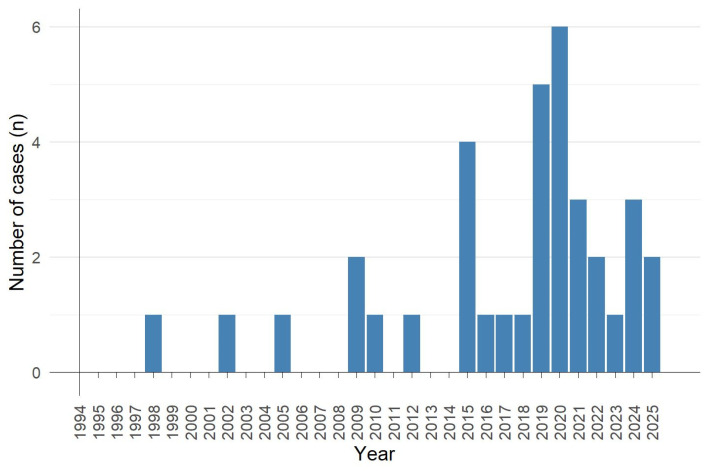
Temporal fluctuations in the number of clinical cases of thyroid hormone poisoning (*n* = 34) over the last 30 years.

The temporal fluctuations in the number of clinical cases of thyroid hormone poisoning over the last 30 years are shown in Figure 2. The first case was published in 1998, and the last one in 2025. Between 1998 and 2008, only three reports of thyroid hormone poisoning were published. The number of cases began to increase in 2009, with at least one case report published annually from 2015 to 2025. The maximum number of clinical cases of thyroid hormone poisoning occurred in 2019 and 2020, with five and six cases reported in each year, respectively. A significant upward trend in the number of cases of thyroid hormone poisoning was observed (Spearman regression: r = 0.66, *p* = 0.0001).

### 3.2. The Cause of Thyroid Hormone Poisoning

The main reasons for thyroid hormone use are presented in Table 1. They were extracted from clinical case texts in the process of data extraction after establishing their definitions. The term “as doping” refers to the use of thyroid hormones by amateur athletes for reasons related to sports practice. The term “in suicide attempts” refers to the use of thyroid hormones for self-harm. The term “to reduce symptoms” refers to the situation of exceeding the doses prescribed by a doctor due to the lack of improvement in treatment with the recommended doses. The term “weight loss” refers to the illegal use of thyroid hormones for weight loss. The term “unknown” refers to clinical studies in which the cause of the overdose of thyroid hormones was not specified. The term “postoperative hypothyroidism” refers to a situation in which the dose of thyroid hormones was exceeded in an attempt to establish replacement therapy. The term “iatrogenic overdose” refers to other situations in which the dose of thyroid hormones was accidentally exceeded because of a doctor’s incorrect recommendation.

The main reasons for thyroid hormone use that led to poisoning included the following: use for doping, suicide attempts, alleviation of hypothyroidism symptoms, and weight loss.

In one case, thyroid hormones were used for doping and for suicide purposes simultaneously. The distribution of individual reasons for thyroid hormone use among the remaining number of clinical cases is presented in Table 1. Approximately 33% of poisoning cases were related to doping in amateur sports (12 cases). Among the cases where thyroid hormones were not used for doping, 63% were suicide attempts (15 cases), approximately 13% entailed the desire to lose weight (three cases), 13% were iatrogenic overdose (3 cases), and approximately 8% entailed the desire to reduce the symptoms of hypothyroidism (2 cases).

Among people using thyroid hormones for doping, in 82% of cases (nine cases), the intended purpose was not specified, and in approximately 18% of cases (two cases) the goal was reducing body fat mass.

Differences between the reasons for using thyroid hormones in the group using them for doping and the group not using them for doping were statistically significant (Fisher’s exact test: *p* = 0.0002).

### 3.3. Temporal and Geographical Variations in Cases of Thyroid Hormone Poisoning

**Figure 3:**
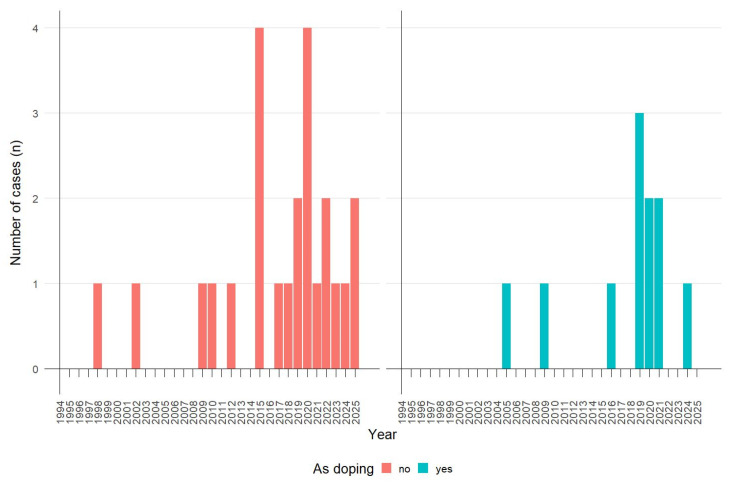
The temporal fluctuations in the number of clinical cases of thyroid hormone poisoning over the last 30 years by reason for use (excluding the case of simultaneous use for doping and suicide purposes). The number of cases: “no”—23; “yes”—10.

The temporal fluctuations in the number of clinical cases of thyroid hormone poisoning over the last 30 years depending on the reason for use are presented in Figure 3.

A statistically significant increase in the number of poisonings associated with the use of these hormones for doping was observed (Spearman regression: r = 0.43, *p* = 0.0265), paralleling a significant increase in the total number of poisonings resulting from the use of thyroid hormones in the years 1995–2025. There was no significant increase in the number of suicide-related poisonings (Spearman regression: r = 0.46, *p* = 0.062).

By continent, the largest number of cases came from North America (ten cases). Seven case reports originated from both Asia and Europe, and two each came from Australia and New Zealand. By country, the largest number of case reports of thyroid hormone poisoning came from the United States (eight cases). The second most common country of origin for clinical case reports of thyroid hormone poisoning was Great Britain (four cases), and the third was China (nine cases).

Table 2 shows the geographical distribution of the reported cases of thyroid hormone poisoning, stratified by the reasons for use.

In the case of thyroid hormone use for doping, most clinical case reports of poisoning come from North America and Europe (five publications each), and one case comes from Asia. In the cases of thyroid hormone use for reasons other than doping, most clinical case reports of poisoning come from Asia (eight publications), six cases each from Europe and North America, and two from Australia and Oceania.

There were no significant differences in geographical variation between case reports of thyroid hormone poisoning when used for doping and for other reasons (Fisher’s exact test: *p* = 0.65).

### 3.4. History of Previous Thyroid Disease or Other Illnesses

In the case of thyroid hormone use for doping, the absence of prior thyroid disease was dominant (Table 3). In use for other purposes, prior thyroid disease was dominant (Table 3). The difference between the groups was statistically significant (Fisher’s exact test: *p* = 0.0233).

The relationships between pre-existing thyroid disease and the reason for using thyroid hormones were statistically significant (Fisher’s exact test: *p* = 0.0131), especially because of the high proportion of the reason “as doping” in the group without pre-existing thyroid hormone disease.

### 3.5. Type of Thyroid Hormone Used in the Clinical Case Reports and Dosage Regimen

Only in one case were T4 and liothyronine (T3) used concurrently. In the context of weight management and use for doping, similar numbers of individuals used T4 and T3, whereas T4 was predominantly utilized in suicide attempt cases. T3 was mainly used to reduce symptoms and in cases of iatrogenic poisoning. The differences were statistically significant (Fisher’s exact test: *p* = 0.0138). The distribution of T4 and T3 in cases of thyroid hormone poisoning is presented in Table 4.

Table 5 presents a summary of poisoning cases resulting from chronic use, acute high-dose use, and acute high-dose use by chronic hormone users.

The most common causes of poisoning were an acute high dose of thyroid hormone or an acute high dose in individuals taking these hormones chronically (approximately 39% each). Cases of poisoning resulting from chronic thyroid hormone use accounted for 19% of poisonings.

There were significant differences in the way thyroid hormones were taken depending on the cause of poisoning (Fisher’s exact test: *p* = 0.0002) (Table 6). In suicide attempts, the predominant pattern was the use of an acute high dose in individuals previously using thyroid hormones chronically. In the cases of using thyroid hormones for doping and for weight loss, the predominant pattern involved the use of an acute high dose without prior treatment. In all cases of poisoning caused by attempts to alleviate symptoms, thyroid hormones were used chronically.

The medians and ranges of doses of individual hormones by the dosing method are presented in Table 7.

### 3.6. Patient Demographic Data

The share of both genders in the total number of thyroid hormone poisonings was similar when the cause of poisoning was not considered (Table 8).

However, statistically significant differences in gender distribution were observed when thyroid hormone poisonings were stratified by cause (Fisher’s exact test: *p* = 0.0026).

Females predominated in cases of suicide attempts, reduction in symptoms, and reduction in body weight, whereas males predominated in doping-related cases (Table 9).

**Figure 4:**
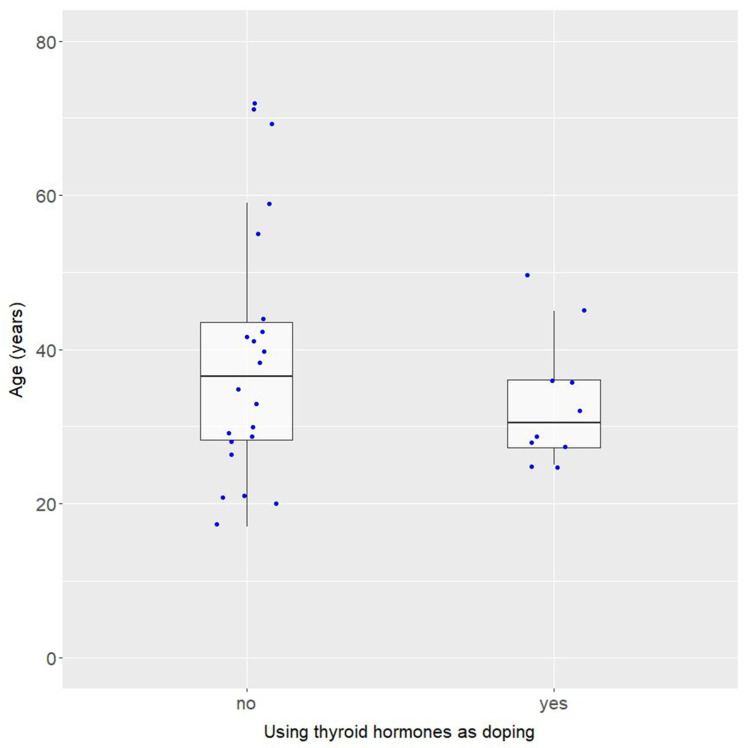
Age of patients depending on whether thyroid hormones were used for doping (*n* = 11) or for another reason (*n* = 23).

Summary statistics of age for all cases are presented in Figure 4.

Although the median ages differed by 6 years, these groups did not differ significantly in terms of age (Wilcoxon test: W = 90.5; *p* = 0.4395). However, the group using thyroid hormones for doping was more homogeneous in terms of age and the difference in variance between these groups was close to statistically significant (Levene’s test for homogeneity of variance: F = 3.21, *p* = 0.0832).

### 3.7. Characteristics of Cases of Poisoning with Thyroid Hormones Used for Doping

The described cases of thyroid hormone poisoning involved bodybuilding. Only one case involved weightlifting.

### 3.8. Substances Used Concurrently with Thyroid Hormones

In most cases of thyroid hormone use for doping, one to nine (median = three) other doping substances were used concurrently. In the one remaining case, the reports did not specify whether thyroid hormones were used concurrently with other doping substances. Among those who used additional doping substances, the majority used multiple substances simultaneously. If only one single additional substance was used, it was testosterone (the data in Supplementary Table S3).

The most frequently used doping substance in conjunction with thyroid hormones was testosterone, followed by trenbolone and clenbuterol. Other drugs used concomitantly with thyroid hormones used for doping included propranolol, propylthiouracil, furosemide, valsartan, bisoprolol, and dapagliflozin (Supplementary Table S4). In four cases of using thyroid hormones for reasons other than for doping, no other drugs were used at the same time. A summary of medicines used concurrently with thyroid hormones not used for doping in individual clinical case reports is presented in Supplementary Table S5. In the case of the use of thyroid hormones for suicidal purposes, psychotropic drugs also dominated among the drugs used. In seven out of nine cases of using thyroid hormones for suicidal purposes together with other drugs, at least one drug from the group of antidepressants, sedatives, or hypnotics was additionally used. A variety of medications were used with thyroid hormones for weight loss or symptom relief.

### 3.9. Clinical Diagnoses Associated with Thyroid Hormone Toxicity

The clinical diagnoses associated with thyroid hormone toxicity are presented in Table 10.

**Figure 5:**
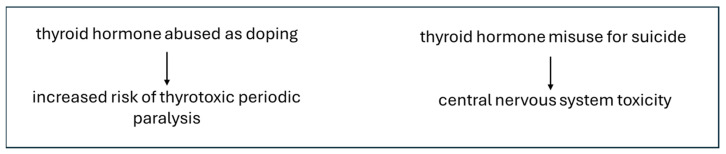
The most characteristic clinical diagnosis depending on the cause of thyroid hormone poisoning.

There were close to statistically significant differences in clinical diagnoses when thyroid hormones were used for doping and for other reasons (Fisher’s exact test: *p* = 0.0964). The most common clinical diagnosis in doping cases was thyrotoxic periodic paralysis, which did not occur in cases where thyroid hormone poisoning occurred for other reasons. Central nervous system symptoms, however, were not observed in doping cases but were associated with thyroid hormone use for other purposes, particularly suicidal use. This is illustrated in Figure 5.

Supplementary Tables S6 and S7 summarize the diagnoses associated with thyroid hormone poisoning cases by the method of administration and the type of drug. Due to the small number of cases, a comparative analysis was not performed.

Of the 34 total cases of thyroid hormone poisoning, 2 were lethal. One was the result of thyroid hormone use for doping, and one was the result of suicide. In both cases, the poisoning was caused by an acute high dose of thyroid hormones (the dose in the suicide attempt was unknown; in the case of use for doping, the dose was 13.5 mg). In both cases, the cause of death was acute heart failure.

## 4. Discussion

Over the past 30 years, 34 clinical cases of thyroid hormone poisoning have been published, all of which involved illegal use of these drugs or off-label doses. This is a relatively small number, especially considering that only two cases resulted in death. However, despite the relatively small number of poisonings with these drugs, there is a statistically significant upward trend in the number of poisonings, which highlights the need to analyze the phenomenon. This could indirectly indicate an upward trend in their abuse, but publication bias, selectivity of case reports, and the increase in the number of journals devoted to case reports must be taken into account.

We showed that thyroid hormone abuse resulted from four main reasons: use for doping purposes, suicide attempts, clinical symptoms of hypothyroidism reduction, and weight loss. Other cases were iatrogenic.

We demonstrated that the majority of individuals using thyroid hormones for doping were male, with a median age of 36.5 years, while half of these individuals were between 28 and 44 years of age. This is consistent with our previous research findings, which indicate that the typical anabolic–androgenic steroid user is a man aged 26–35 years [18]. On the other hand, in the case of the use of thyroid hormones for suicide purposes, the female gender was dominant, which is consistent with the fact that females have higher rates of suicide attempts than males [50].

There was no territorial differentiation in cases of thyroid hormone use for doping, which is also consistent with our previous research results in a study on using anabolic–androgenic steroids [51]. Although globally, most suicide attempters live in low- and middle-income countries, our study did not demonstrate any territorial variation in the use of thyroid hormones for suicide. This may have been caused by the small number of cases [52].

The relation between the choice of thyroid hormone and the cause of its abuse is an interesting issue. According to the standard treatment for hypothyroidism, T4 is the first-line drug [53]. Combination therapy with T4 and T3 should be considered only as an experimental treatment method [54]. For this reason, T4 preparations are more readily available. This greater availability of the drug may explain why thyroid hormone abuse primarily involves T4. Misuse of this drug predominates in suicide attempts. In more than 50% of cases of poisoning with suicidal intent, patients had a history of thyroid disease treated with T4 and therefore had access to the drug. In 100% of the cases of poisoning caused by attempts to reduce the symptoms of hypothyroidism, T3 was used, which likely resulted from T4 ineffectiveness. When hormones are used for doping purposes, the share of T4 and T3 is similar. Most individuals engaged in doping also use other performance-enhancing substances, which can only come from accessible illegal sources. In this case, one can speculate that thyroid hormones are also obtained illegally, and thus the commonness of the drug is not the main factor determining its choice. When analyzing the type of drug used in correlation with the purpose of its use, the small sample size should, of course, be taken into account.

Most cases of thyroid hormone toxicity resulted from the administration of an acute high dose of the drug in previously untreated individuals or an acute high dose in previously treated individuals. Both lethal cases were caused by an acute high dose in previously untreated individuals. This indicates that chronic use of lower doses was less likely to cause toxicity symptoms and was not fatal in any case. Our observations may not reflect the actual pattern of illicit thyroid hormone use due to publication bias. In this study, we only analyzed cases of acute poisoning.

The significant increase in the overall number of thyroid hormone poisoning cases is accompanied by a parallel rise in cases related to doping. This upward trend in thyroid hormone poisoning for doping reflects the increasing use of anabolic–androgenic steroids, which can be observed by analyzing the results of studies on the frequency of illicit anabolic–androgenic steroid use over the years. A meta-analysis of 271 articles by Sagoe et al. showed that 18.4% of recreational athletes had used androgens during their lifetime [55]. Similar results were obtained by Alsaeed et al. (22.7% of 194 respondents) in a cross-sectional study of ten fitness clubs in Kuwait [56]. Both studies were from the years 2014–2015. In two subsequent studies by Montuori et al. and Skrzypiec-Spring et al. published in 2021 and 2024, an increase in the percentage of amateur athletes using anabolic–androgenic steroids to approximately 35% was noted [18,57]. Moreover, our previous work demonstrated an upward trend in the number of anabolic–androgenic steroid poisonings [51].

It is believed that thyroid hormones are usually used in doping as an addition to anabolic–androgenic steroids, either to reduce the amount of body fat or to compensate for hormonal imbalances induced by anabolic–androgenic steroid use [58]. This has been confirmed by the results of our study, as in most cases of thyroid hormone poisoning when used for doping, at least one other performance-enhancing substance was used concurrently. In all cases of combined use, when only one additional substance was used, it was testosterone. However, in most cases, multiple substances were used simultaneously, usually 3–4 substances.

In most cases of thyroid hormone use for doping, anabolic–androgenic steroids were used concurrently. Therefore, one can postulate that symptoms of thyroid hormone toxicity in these cases may have resulted from the overlap of adverse effects of all the abused substances. The most common clinical diagnoses in these individuals were thyrotoxic periodic paralysis and heart failure. Other diagnoses included atrial fibrillation, cardiomyopathy, and myocardial infarction. Thyrotoxic periodic paralysis is believed to result from excessive stimulation of Na-K-ATPase [59]. This leads to an influx of potassium into skeletal muscle, resulting in a disruption of the skeletal muscle rectifier potassium channel and paradoxical depolarization, subsequent inactivation of sodium channels, and paralysis [59]. All cases of thyrotoxic periodic paralysis occurred in individuals using thyroid hormones as doping agents. The authors believe that there is a link between a predisposition to thyrotoxic periodic paralysis resulting from thyroid hormone poisoning and the use of anabolic–androgenic steroids or increased physical exercise in these individuals. No data on this association currently exist in the literature. This association stems from the increased muscle mass in individuals using anabolic–androgenic steroids. However, there is also a possible link between the increased Na^+^, K^+^-ATPase activity and intense physical exercise itself [60,61,62]. In our previous work, we showed that among the various types of diseases resulting from the use of anabolic–androgenic steroids over a 50-year period, the most common were cardiovascular diseases, with myocardial infarction dominating, and hypertrophic and dilated cardiomyopathy in the second and third places [51]. Thyrotoxicosis increases the heart’s oxygen demand through upregulation of beta receptors, leading to elevated heart rate, stroke volume, and cardiac contractility. Therefore, it is possible that increased oxygen demand caused by thyroid hormone overdose in heart muscle exposed to the adverse effects of anabolic–androgenic steroids may potentiate the cardiotoxicity of anabolic–androgenic steroids. Interestingly, none of the individuals poisoned with thyroid hormones as a result of doping had central nervous system symptoms as the primary diagnosis. However, central nervous system symptoms, such as delirium, seizures, coma, and psychosis, occurred in over half of the individuals whose thyroid hormone poisoning occurred for reasons other than use for doping. Among these individuals, over half were taking psychotropic medications, such as benzodiazepines, tricyclic antidepressants, and selective serotonin reuptake inhibitors, which may indicate a link between the use of these medications and the manifestations of thyroid hormone poisoning in the central nervous system. The effect of thyroid hormones on multiple components of the gamma aminobutyric acid (GABA) system has been demonstrated, although this issue is not fully understood [63]. Moreover, relationships between thyroid hormones and the brain’s serotonin system have also been demonstrated [64].

Despite the correlations we observed between the types of complications of thyroid hormone poisoning and the purpose of use, it is necessary to take into account the limitation of the small number of publications and the fact that some cases with a milder course may not be published as clinical case reports.

Thyroid hormone abuse occurs due to a combination of factors, including the mechanism of action, availability, and low price of these hormones. Their overuse can lead to serious health consequences, including death. These medications are used to treat the common condition of hypothyroidism, and are considered safe in therapeutic doses. Therefore, in many countries, it is possible to obtain a prescription for these medications online, facilitating access. Given the growing trend of misusing these medications for doping and their persistent abuse for weight loss and suicide, which has been constant over the past few years, determined efforts should be made to reduce the availability of these medications online by introducing appropriate legal regulations. Like anabolic–androgenic steroids, thyroid hormones are also available on the black market, where medications often have unknown compositions and dosages, which can lead to unpredictable effects. Therefore, it is necessary to strive to increase control over the illegal black-market distribution of medicines and other abused substances through legal regulations.

Our study is the first attempt to assess and characterize the consequences of thyroid hormone abuse in the form of drug poisoning. The limitations of this study stem from the small number of reported cases, which complicates statistical analysis, heterogeneous dataset, publications only in English, and strong confounding by co-occurring substances. Furthermore, the number of cases described in the literature is certainly lower than the actual number of thyroid hormone poisonings because many cases remain undocumented or unpublished in official clinical or toxicological reports.

Nevertheless, despite these limitations, our research provides a valuable summary of the facts regarding thyroid hormone poisoning and indicates a growing trend in this type of poisoning.

## 5. Conclusions

The number of clinical cases describing acute thyroid hormone poisoning has shown an upward trend over the past 30 years, particularly since 2015. The same dynamics were observed in the increase in clinical cases of thyroid hormone poisoning used for doping purposes. Thyroid hormones are typically used as an adjunct to anabolic–androgenic steroids and may exacerbate the heart damage they cause. This study has proven that anabolic–androgenic steroids and/or intense strength training appear to increase the risk of a rare condition called thyrotoxic periodic paralysis. The number of cases of thyroid hormone use in suicide attempts since 2017 has remained stable, indicating that thyroid hormone abuse is also an important issue in suicidology. However, the authors would like to highlight the limitations of this manuscript, namely the small, heterogeneous dataset, publications only in English, and strong confounding by co-occurring substances, which should be taken into account when drawing conclusions. Despite its limitations, the manuscript indicates the need to prevent the abuse of thyroid hormones by introducing legal regulations that would make it difficult to obtain prescriptions for drugs online and to better control their illegal online distribution. It also signals the necessity of launching information campaigns that would increase public awareness of the harmful effects of using medications without medical indications.

## Figures and Tables

**Table 1:** The main reasons for thyroid hormone use, excluding the case of simultaneous use for doping and for suicide purposes. *n*—number of cases; %—percentage of cases.

Reasons for Using a Thyroid-Related Drug	For Doping		Other Reasons		Total	
	n	%	n	%	n	%
suicide	0	0	15	63	15	43
to reduce symptoms	0	0	2	8	2	6
unknown	9	82	0	0	9	26
weight loss	2	18	3	13	5	14
postoperative hypothyroidism	0	0	1	4	1	3
iatrogenic overdose	0	0	3	13	3	9
Total	11	100	24	100	35	100

**Table 2:** The geographical distribution of the reported cases of thyroid hormone poisoning, stratified by the reasons for use (excluding the case of simultaneous use for doping and for suicide purposes). *n*—number of cases; %—percentage of cases.

Continent	For Doping		Other Reasons		Total	
	n	%	n	%	n	%
Asia	1	9	8	36	9	26
Australia and Oceania	0	0	2	9	2	6
Europe	5	45	6	27	11	31
North America	5	45	6	27	13	37
Total	11	100	22	100	35	100

**Table 3:** The relationships between a pre-existing thyroid disease and the reason for using thyroid hormones when stratified by the reasons for their use (excluding the case of simultaneous use for doping and suicide purposes). *n*—number of cases; %—percentage of cases.

Pre-Existing Thyroid Disease	For Doping		Other								Total	
			Iatrogenic		Suicide		To Reduce Symptoms		Weight Loss			
	n	%	n	%	n	%	n	%	n	%	n	%
No	10	48	1	5	8	38	0	0	2	10	21	100
Yes	1	7	3	21	6	43	3	21	1	7	14	100

**Table 4:** Distribution of T4 and T3 in cases of thyroid hormone poisoning presented by the cause (excluding the case of simultaneous use for doping and for suicide purposes). *n*—number of cases; %—percentage of cases.

Reasons for Using a Thyroid-Related Drug	Compounded T3/T4		T4		T3		Unknown		Total	
	n	%	n	%	n	%	n	%	n	%
For doping	0	0	5	45	4	36	2	18	11	100
Iatrogenic	1	25	1	25	2	50	0	0	4	100
Suicide	0	0	13	93	1	7	0	0	14	100
To reduce symptoms	0	0	1	33	2	67	0	0	3	100
Weight loss	0	0	1	33	1	33	1	33	3	100

**Table 5:** Summary of poisoning cases resulting from chronic use, acute high-dose use, and acute high-dose use by chronic hormone users. *n*—number of cases; %—percentage of cases.

Way of Using	n	%
Chronic	7	19
Chronic and acute high dose	14	39
Acute high dose	14	39
Other	1	3
Total	36	100

**Table 6:** The way of taking thyroid hormones presented by the cause of poisoning (excluding the case of simultaneous use for doping and for suicide purposes). *n*—number of cases; %—percentage of cases.

Reasons for Using a Thyroid-Related Drug	Chronic		Chronic and One Acute High Dose		Acute High Dose		Other		Total	
	n	%	n	%	n	%	n	%	n	%
For doping	2	18	1	9	7	64	1	9	11	100
Iatrogenic	1	25	3	75	0	0	0	0	4	100
Suicide	0	0	10	71	4	29	0	0	14	100
To reduce symptoms	3	100	0	0	0	0	0	0	3	100
Weight loss	1	33	0	0	2	67	0	0	3	100

**Table 7:** Medians and dose ranges of individual hormones by the dosing method. *n*—number of cases. Min—minimum dose; Max—maximum dose. Median, Min, and Max values are expressed in milligrams.

Thyroid_Related_Medication	Consumption_Type	N	Median	Min	Max
Levothyroxine	Chronic use	3	0.100	0.050	0.150
	Chronic use and acute high dose	6	0.100	0.006	0.150
	Other	1	0.200	0.200	0.200
Liothyronine	Chronic use	2	0.025	0.025	0.025
	Chronic use and acute high dose	3	10.744	0.075	15.000
	Acute high dose	4	0.050	0.025	0.075

**Table 8:** The number of thyroid hormone poisoning cases by gender. *n*—number of cases; %—percentage of cases.

Sex	n	%
Females	21	58
Males	15	42

**Table 9:** The distribution of gender in thyroid hormone poisoning by the cause of poisoning. *n*—number of cases; %—percentage of cases.

Sex	For Doping		Iatrogenic		Suicide		To Reduce Symptoms		Weight Loss	
	n	%	n	%	n	%	n	%	n	%
Females	2	18	2	50	12	86	3	100	2	67
Males	9	82	2	50	2	14	0	0	1	33
Total	11	100	4	100	14	100	3	100	3	100

**Table 10:** Summary of diagnoses associated with thyroid hormone poisoning cases in the group of people using them for doping and for other reasons. *n*—number of cases; %—percentage of cases.

Diagnosis	Thyroid Hormones Used for Doping		Thyroid Hormones Used for Other Reasons	
	n	%	n	%
Thyrotoxic periodic paralysis	4	40	0	0
Tachycardia	2	20	7	30
St-elevation myocardial infarction (STEMI)	1	10	1	4
Heart failure	1	10	1	4
Cardiogenic shock	1	10	0	0
Cardiomyopathy	1	10	0	0
Coma	0	0	2	9
Atrial fibrillation	0	0	2	9
Delirium	0	0	2	9
No symptoms of thyroid toxicity	0	0	2	9
Seizure	0	0	2	9
Dyspnea	0	0	1	4
Psychosis	0	0	1	4
Thyrotoxicosis	0	0	1	4
Tremor	0	0	1	4
Total	10	100	23	100

## Data Availability

All data generated or analyzed during this study are included in this published article.

## Supplementary Materials

The following supporting information can be downloaded at https://www.mdpi.com/article/10.3390/ph18121808/s1. Table S1. PRISMA 2020 checklist. Table S2. List of clinical cases of thyroid hormone poisoning. And summary of main outcome. Table S3. Summary of other substances used concurrently with thyroid hormones as doping in individual clinical case reports. Table S4. Summary of medicines used concurrently with thyroid hormones as doping in individual clinical case reports. Table S5. Summary of medicines used concurrently with thyroid hormones not used as doping in individual clinical case reports. Table S6. The number of thyroid hormone poisoning cases depending on how they were used. Table S7. The number of thyroid hormone poisoning cases by drug type.

## Abbreviations

The following abbreviations are used in this manuscript:

WADA	World Anti-Doping Agency
GABA	gamma aminobutyric acid
STEMI	ST-elevation myocardial infarction

## References

[1] Quittkat H.L., Hartmann A.S., Düsing R., Buhlmann U., Vocks S. (2019). Body Dissatisfaction, Importance of Appearance, and Body Appreciation in Men and Women Over the Lifespan. Front. Psychiatry.

[2] Barbierik L., Bacikova-Sleskova M., Petrovova V. (2023). The Role of Social Appearance Comparison in Body Dissatisfaction of Adolescent Boys and Girls. Eur. J. Psychol..

[3] Kim B., Carvalho-Bianco S.D., Larsen P.R. (2004). Thyroid hormone and adrenergic signaling in the heart. Arq. Bras. Endocrinol. Metabol..

[4] Harper M.-E., Seifert E.L. (2008). Thyroid hormone effects on mitochondrial energetics. Thyroid..

[5] Mullur R., Liu Y.-Y., Brent G.A. (2014). Thyroid hormone regulation of metabolism. Physiol. Rev..

[6] Cicatiello A.G., di Girolamo D., Dentice M. (2018). Metabolic effects of the intracellular regulation of thyroid hormone: Old players, new concepts. Front. Endocrinol..

[7] Salvatore D., Simonides W.S., Dentice M., Zavacki A.M., Larsen P.R. (2014). Thyroid hormones and skeletal muscle—New insights and potential implications. Nat. Rev. Endocrinol..

[8] Nappi A., Moriello C., Morgante M., Fusco F., Crocetto F., Miro C. (2024). Effects of thyroid hormones in skeletal muscle protein turnover. J. Basic. Clin. Physiol. Pharmacol..

[9] Santini F., Marzullo P., Rotondi M., Ceccarini G., Pagano L., Ippolito S., Chiovato L., Biondi B. (2014). The crosstalk between thyroid gland and adipose tissue: Signal integration in health and disease. Eur. J. Endocrinol..

[10] Martínez Brito D., Leogrande P., de la Torre X., Botrè F. (2025). Detection of thyroid hormones in urine by liquid chromatography coupled to tandem mass spectrometry. Drug Test. Anal..

[11] Galofré J.C., Díez J.J., Attanasio R., Nagy E.V., Negro R., Papini E., Perros P., Žarković M., Akarsu E., Alevizaki M. (2024). Treatment of obesity with thyroid hormones in Europe. Data from the THESIS* Collaboration. J. Endocrinol. Investig..

[12] Toloza F.J.K., Mao Y., Menon L., George G., Borikar M., Thumma S., Motahari H., Erwin P., Owen R., Maraka S. (2021). Association of thyroid function with suicidal behavior: A systematic review and meta-analysis. Medicina.

[13] World Anti-Doping Agency World Anti-Doping Code. https://www.wada-ama.org/en/resources/world-anti-doping-program/world-anti-doping-code.

[14] Pokrywka A., Surała O., Grabowska K., Przybyła M., Granda D., Małecki A., Faiss R., Nowacka-Chmielewska M. (2025). “Brain doping” substances: Prohibited or not in sports?. Biol. Sport.

[15] Handelsman D.J., Gild M., Clifton-Bligh R., Speers N., Kouzios D., McMartin M.C., Desai R. (2023). Thyroid hormone abuse among elite athletes. J. Endocr. Soc..

[16] Gild M.L., Stuart M., Clifton-Bligh R.J., Kinahan A., Handelsman D.J. (2022). Thyroid hormone abuse in elite sports: The regulatory challenge. J. Clin. Endocrinol. Metab..

[17] McBride J.A., Carson C.C., Coward R.M. (2018). The Availability and Acquisition of Illicit Anabolic Androgenic Steroids and Testosterone Preparations on the Internet. Am. J. Mens. Health.

[18] Skrzypiec-Spring M., Pokrywka A., Bombała W., Berezovska D., Rozmus J., Brawańska K., Nowicki K., Abu Faraj G., Rynkowski M., Szeląg A. (2024). Illegal use of testosterone and other anabolic–androgenic steroids in the population of amateur athletes in Wrocław, Poland—An unfavorable lifestyle trend in the population of men of reproductive age. J. Clin. Med..

[19] Page M.J., McKenzie J.E., Bossuyt P.M., Boutron I., Hoffmann T.C., Mulrow C.D., Shamseer L., Tetzlaff J.M., Akl E.A., Brennan S. (2021). The PRISMA 2020 statement: An updated guideline for reporting systematic reviews. BMJ.

[20] Ishihara T., Nishikawa M., Ikekubo K., Kajikawa M., Kobayashi H., Hino M., Moridera K., Kasagi K., Inada M., Kurahachi H. (1998). Thyroxine (T4) Metabolism in an Athyreotic Patient Who Had Taken a Large Amount of T4 at One Time. Endocr. J..

[21] Mark P.B., Watkins S., Dargie H.J. (2005). Cardiomyopathy Induced by Performance Enhancing Drugs in a Competitive Bodybuilder. Heart.

[22] Chen Y.-C., Fang J.-T., Chang C.-T., Chou H.-H. (2001). Thyrotoxic Periodic Paralysis in a Patient Abusing Thyroxine for Weight Reduction. Ren. Fail..

[23] da Silva J.A., Almeida J.T., Corrêa B.B., Narigão M., Xavier M. (2009). Acute Psychotic Episode in a Patient with Thyrotoxicosis Factitia. BMJ Case Rep..

[24] Hartung B., Schott M., Daldrup T., Ritz-Timme S. (2010). Lethal Thyroid Storm after Uncontrolled Intake of Liothyronine in Order to Lose Weight. Int. J. Leg. Med..

[25] Cantrell L. (2012). Redotex® Revisited: Intentional Overdose with an Illegal Weight Loss Product. J. Emerg. Med..

[26] Allen K.M., Crawford V.B., Conaglen J.V., Elston M.S. (2015). Case Report: Clues to the Diagnosis of an Unsuspected Massive Levothyroxine Overdose. Can. J. Emerg. Med..

[27] Bains A., Brosseau A.-J., Harrison D. (2015). Iatrogenic Thyrotoxicosis Secondary to Compounded Liothyronine. Can. J. Hosp. Pharm..

[28] Rothberger G.D., Desai A.K., Sharif S., Chawla S.A., Shirazian S. (2015). The Case: Elevated Lactate and Osmolar Gap after Levothyroxine Overdose. Kidney Int..

[29] Kwak T., Al Zoubi M., Bhavith A., Rueda Rios C., Kumar S. (2016). Acute Myocarditis in Bodybuilder from Coxsackievirus and Thyrotoxicosis. J. Cardiol. Cases.

[30] D’Arcy R., McDonnell M., Spence K., Courtney C.H. (2017). Exogenous T3 Toxicosis Following Consumption of a Contaminated Weight Loss Supplement. Endocrinol. Diabetes Metab. Case Rep..

[31] Xue J., Zhang L., Qin Z., Li R., Wang Y., Zhu K., Li X., Gao X., Zhang J. (2018). No Obvious Sympathetic Excitation after Massive Levothyroxine Overdose. Medicine.

[32] Roomi S., Ullah W., Iqbal I., Ahmad A., Saleem S., Sattar Z. (2019). Thyrotoxicosis Factitia: A Rare Cause of Junctional Rhythm and Cardiac Arrest. J. Community Hosp. Intern. Med. Perspect..

[33] Daher G., Hassanieh I., Malhotra N., Alderson L. (2019). Acute Decompensated Heart Failure Secondary to Exogenous Triiodothyronine Use in a Young Non-Athlete Weightlifter. Cureus.

[34] Wong O., Wong A., Greene S., Graudins A. (2018). Prolonged Coma Resulting from Massive Levothyroxine Overdose and the Utility of NT-proBNP. Clin. Toxicol..

[35] Warner B.E., Woodrow C.J., Pal A. (2020). Delayed Diagnosis of T3 Supplementation in a Bodybuilder Presenting with Tachycardia and Features of Sepsis. BMJ Case Rep..

[36] Patel A.J., Tejera S., Klek S.P., Rothberger G.D. (2020). Thyrotoxic Periodic Paralysis in a Competitive Bodybuilder with Thyrotoxicosis Factitia. AACE Clin. Case Rep..

[37] Kiran Kumar K.C., Ghimire N., Limbu T., Khapung R. (2020). Levothyroxine Overdose in a Hypothyroid Patient with Adjustment Disorder: A Case Report. Ann. Med. Surg..

[38] van Bokhorst Q., Krul-Poel Y., Smit D., de Ronde W. (2021). A 29-Year-Old Bodybuilder with Liothyronine-Induced Thyrotoxic Hypokalaemic Periodic Paralysis. Eur. J. Case Rep. Intern. Med..

[39] Bonnar C.E., Brazil J.F., Okiro J.O., Giblin L., Smyth Y., O’Shea P.M., Finucane F.M. (2021). Making Weight: Acute Muscle Weakness and Hypokalaemia Exacerbated by Thyrotoxicosis Factitia in a Bodybuilder. Endocrinol. Diabetes Metab. Case Rep..

[40] Du F., Liu S.-W., Yang H., Duan R.-X., Ren W.-X. (2022). Thyrotoxicosis after a Massive Levothyroxine Ingestion: A Case Report. World J. Clin. Cases.

[41] Gill A.S., Rai H.K., Karunakaran A., Chaudhuri A. (2023). Suicide Attempt With Levothyroxine Overdose. Cureus.

[42] Momoh R., Hassan A. (2024). A Case Report of an Acute Severe Tachyarrhythmia with Underlying Cardiomyopathy in a Patient with Anabolic Androgenic Steroid and Thyroxine Misuse. Cureus.

[43] Sowmya D., Subhasri G., Dixit A., Katam S., Odoch A.K., Jeyasundar D., Hassan M., Sowah A.O., Akwue T.A., Tilahun W.T. (2024). Acute MI of Young Age: Unwarranted Dosing of Steroidal and Nutritional Supplementations. Am. J. Intern. Med..

[44] Theocharidou C.-C., Pavlidou M., Endiaroglou M., Dimaki A., Ampatzidou F. (2025). Coma as the Sole Initial Manifestation of Levothyroxine Intoxication: A Case Report. J. Emerg. Med..

[45] He Z.H., Li Y., Trivedi N., Gill S., Hennessey J.V. (2020). Thyrotoxicosis after Massive Triiodothyronine (LT3) Overdose: A Coast-to-Coast Case Series and Review. Drugs Context.

[46] Vorasart P., Sriphrapradang C. (2019). Factitious Thyrotoxicosis: How to Find It. Diagnosis.

[47] Nanjappa H., Rodrigues A.J. (2024). Atrial Fibrillation Secondary to Levothyroxine Overdose with Underlying Secondary Infection. J. Assoc. Physicians India.

[48] Li R., Xu Y.-W., Xue Y., Wu X.-Z. (2020). Plasmapheresis in the Treatment of Multi-Drug Intoxication Involving Levothyroxine Sodium and Calcium Channel Blockers: A Case Report. Ann. Palliat. Med..

[49] de Luis D.A., Dueñas A., Martin J., Abad L., Cuellar L., Aller R. (2002). Light Symptoms Following a High-Dose Intentional L-Thyroxine Ingestion Treated with Cholestyramine. Horm. Res. Paediatr..

[50] Berardelli I., Rogante E., Sarubbi S., Erbuto D., Cifrodelli M., Concolato C., Pasquini M., Lester D., Innamorati M., Pompili M. (2022). Is lethality different between males and females? Clinical and gender differences in inpatient suicide attempters. Int. J. Environ. Res. Public Health.

[51] Skrzypiec-Spring M., Rozmus J., Abu Faraj G., Brawańska-Maśluch K., Kujawa K., Szeląg A. (2024). Abuse of anabolic-androgenic steroids as a social phenomenon and medical problem—Its potential negative impact on reproductive health based on 50 years of case report analysis. J. Clin. Med..

[52] World Health Organization (2025). Suicide Worldwide in 2021: Global Health Estimates.

[53] Centanni M., Duntas L., Feldt-Rasmussen U., Koehrle J., Peeters R.P., Razvi S., Trimboli P., Virili C. (2025). ETA guidelines for the use of levothyroxine sodium preparations in monotherapy to optimize the treatment of hypothyroidism. Eur. Thyroid. J..

[54] Wiersinga W.M., Duntas L., Fadeyev V., Nygaard B., Vanderpump M.P.J. (2012). 2012 ETA guidelines: The use of L-T4 + L-T3 in the treatment of hypothyroidism. Eur. Thyroid J..

[55] Sagoe D., Molde H., Andreassen C.S., Torsheim T., Pallesen S. (2014). The global epidemiology of anabolic-androgenic steroid use: A meta-analysis and meta-regression analysis. Ann. Epidemiol..

[56] Alsaeed I., Alabkal J.R. (2015). Usage and perceptions of anabolic-androgenic steroids among male fitness centre attendees in Kuwait: A cross-sectional study. Subst. Abus. Treat. Prev. Policy.

[57] Montuori P., Loperto I., Paolo C., Castrianni D., Nubi R., de Rosa E., Palladino R., Triassi M. (2021). Bodybuilding, dietary supplements and hormones use: Behaviour and determinant analysis in young bodybuilders. BMC Sports Sci. Med. Rehabil..

[58] Mohammed H.H., Owada N.O., Mohammed N.J. (2024). Thyroid hormone use in athletes: Physiological insights and doping controversies. J. Shifa Tameer-E-Millat Univ..

[59] Lin S.-H., Huang C.-L. (2012). Mechanism of thyrotoxic periodic paralysis. J. Am. Soc. Nephrol..

[60] Evertsen F., Medbo J.I., Jebens E., Nicolaysen K. (1997). Hard training for 5 mo increases Na(+)-K+ pump concentration in skeletal muscle of cross-country skiers. Am. J. Physiol.-Regul. Integr. Comp. Physiol..

[61] Green H.J., Chin E.R., Ball-Burnett M., Ranney D. (1993). Increases in human skeletal muscle Na(+)-K(+)-ATPase concentration with short-term training. Am. J. Physiol.-Cell Physiol..

[62] McKenna M.J., Schmidt T.A., Hargreaves M., Cameron L., Skinner S.L., Kjeldsen K. (1993). Sprint training increases human skeletal muscle Na(+)-K(+)-ATPase concentration and improves K+ regulation. J. Appl. Physiol..

[63] Wiens S.C., Trudeau V.L. (2006). Thyroid hormone and γ-aminobutyric acid (GABA) interactions in neuroendocrine systems. Comp. Biochem. Physiol. A Mol. Integr. Physiol..

[64] Bauer M., Heinz A., Whybrow P.C. (2002). Thyroid hormones, serotonin and mood: Of synergy and significance in the adult brain. Mol. Psychiatry.

