# Supplementary material for: Poisoning with Thyroid Hormones Used Illegally—Systematic Review
Source: Pharmaceuticals (Basel). 2025 Nov 27;18(12):1808. doi: 10.3390/ph18121808 (PMC12736065; doi:10.3390/ph18121808)
Supplement: Supplementary file 1 [file pharmaceuticals-18-01808-s001.zip › pharmaceuticals-3898491-supplementary.pdf]

Supplementary Table S1. PRISMA 2020 checklist

Section and Topic	Item #	Checklist item	Location where item is reported
**TITLE**			
Title	1	Identify the report as a systematic review.	See Title
**ABSTRACT**			
Abstract	2	See the PRISMA 2020 for Abstracts checklist.	See Abstract, Page 1.
**INTRODUCTION**			
Rationale	3	Describe the rationale for the review in the context of existing knowledge.	Page 3
Objectives	4	Provide an explicit statement of the objective(s) or question(s) the review addresses.	Page 3
**METHODS**			
Eligibility criteria	5	Specify the inclusion and exclusion criteria for the review and how studies were grouped for the syntheses.	Page 4
Information sources	6	Specify all databases, registers, websites, organisations, reference lists and other sources searched or consulted to identify studies. Specify the date when each source was last searched or consulted.	Page 4
Search strategy	7	Present the full search strategies for all databases, registers and websites, including any filters and limits used.	Page 4
Selection process	8	Specify the methods used to decide whether a study met the inclusion criteria of the review, including how many reviewers screened each record and each report retrieved, whether they worked independently, and if applicable, details of automation tools used in the process.	Page 4
Data collection process	9	Specify the methods used to collect data from reports, including how many reviewers collected data from each report, whether they worked independently, any processes for obtaining or confirming data from study investigators, and if applicable, details of automation tools used in the process.	Page 4
Data items	10a	List and define all outcomes for which data were sought. Specify whether all results that were compatible with each outcome domain in each study were sought (e.g. for all measures, time points, analyses), and if not, the methods used to decide which results to collect.	Page 4
	10b	List and define all other variables for which data were sought (e.g. participant and intervention characteristics, funding sources). Describe any assumptions made about any missing or unclear information.	Page 4
Study risk of bias assessment	11	Specify the methods used to assess risk of bias in the included studies, including details of the tool(s) used, how many reviewers assessed each study and whether they worked independently, and if applicable, details of automation tools used in the process.	Page 4
Effect measures	12	Specify for each outcome the effect measure(s) (e.g. risk ratio, mean difference) used in the synthesis or presentation of results.	Page 4
Synthesis methods	13a	Describe the processes used to decide which studies were eligible for each synthesis (e.g. tabulating the study intervention characteristics and comparing against the planned groups for each synthesis (item #5)).	Page 4
	13b	Describe any methods required to prepare the data for presentation or synthesis, such as handling of missing summary statistics, or data conversions.	Page 4
	13c	Describe any methods used to tabulate or visually display results of individual studies and syntheses.	Page 4

	13d	Describe any methods used to synthesize results and provide a rationale for the choice(s). If meta-analysis was performed, describe the model(s), method(s) to identify the presence and extent of statistical heterogeneity, and software package(s) used.	Page 4-6
	13e	Describe any methods used to explore possible causes of heterogeneity among study results (e.g. subgroup analysis, meta-regression).	Page 5-6
	13f	Describe any sensitivity analyses conducted to assess robustness of the synthesized results.	Page 5-6
Reporting bias assessment	14	Describe any methods used to assess risk of bias due to missing results in a synthesis (arising from reporting biases).	Page 4
Certainty assessment	15	Describe any methods used to assess certainty (or confidence) in the body of evidence for an outcome.	Page 4
**RESULTS**			
Study selection	16a	Describe the results of the search and selection process, from the number of records identified in the search to the number of studies included in the review, ideally using a flow diagram.	Figure 1, Page 5
	16b	Cite studies that might appear to meet the inclusion criteria, but which were excluded, and explain why they were excluded.	Page 4
Study characteristics	17	Cite each included study and present its characteristics.	Supplementary Table 1
Risk of bias in studies	18	Present assessments of risk of bias for each included study.	Page 4
Results of individual studies	19	For all outcomes, present, for each study: (a) summary statistics for each group (where appropriate) and (b) an effect estimate and its precision (e.g. confidence/credible interval), ideally using structured tables or plots.	Not applicable
Results of syntheses	20a	For each synthesis, briefly summarise the characteristics and risk of bias among contributing studies.	Not applicable
	20b	Present results of all statistical syntheses conducted. If meta-analysis was done, present for each the summary estimate and its precision (e.g. confidence/credible interval) and measures of statistical heterogeneity. If comparing groups, describe the direction of the effect.	Pages 6-15
	20c	Present results of all investigations of possible causes of heterogeneity among study results.	Pages 6-15
	20d	Present results of all sensitivity analyses conducted to assess the robustness of the synthesized results.	Pages 6-15
Reporting biases	21	Present assessments of risk of bias due to missing results (arising from reporting biases) for each synthesis assessed.	Pages 6-15
Certainty of evidence	22	Present assessments of certainty (or confidence) in the body of evidence for each outcome assessed.	Pages 6-16
**DISCUSSION**			
Discussion	23a	Provide a general interpretation of the results in the context of other evidence.	Pages 15-20
	23b	Discuss any limitations of the evidence included in the review.	Pages 15-20
	23c	Discuss any limitations of the review processes used.	Pages 15-20
	23d	Discuss implications of the results for practice, policy, and future research.	Pages 15-20
**OTHER INFORMATION**			
Registration and protocol	24a	Provide registration information for the review, including register name and registration number, or state that the review was not registered.	Not registered
	24b	Indicate where the review protocol can be accessed, or state that a protocol was not prepared.	Not prepared

	24c	Describe and explain any amendments to information provided at registration or in the protocol.	Not prepared
Support	25	Describe sources of financial or non-financial support for the review, and the role of the funders or sponsors in the review.	No external founding
Competing interests	26	Declare any competing interests of review authors.	No conflict of interest
Availability of data, code and other materials	27	Report which of the following are publicly available and where they can be found: template data collection forms; data extracted from included studies; data used for all analyses; analytic code; any other materials used in the review.	In manuscript

From: Page MJ, McKenzie JE, Bossuyt PM, Boutron I, Hoffmann TC, Mulrow CD, et al. The PRISMA 2020 statement: an updated guideline for reporting systematic reviews. BMJ 2021;372:n71. doi: 10.1136/bmj.n71. This work is licensed under CC BY 4.0. To view a copy of this license, visit https://creativecommons.org/licenses/by/4.0/

Supplementary Table S2. List of clinical cases of thyroid hormone poisoning. And summary of main outcome.

Case ID	Publication	Main outcome	Reference
1	Ishihara, T.; Nishikawa, M.; Ikekubo, K.; Kajikawa, M.; Kobayashi, H.; Hino, M.; Moridera, K.; Kasagi, K.; Inada, M.; Kurahachi, H. Thyroxine (T4) Metabolism in an Athyreotic Patient Who Had Taken a Large Amount of T4 at One Time. Endocr J, 1998, 45 (3), 371–375. https://doi.org/10.1507/endocrj.45.371 https://doi.org/10.1081/jdi-100001294.	Levothyroixne was taken in acute high dose to commit suicide and there were no symptoms of thyroid toxicity. Intoxication was not lethal.	20
2	Mark, P. B.; Watkins, S.; Dargie, H. J. Cardiomyopathy Induced by Performance Enhancing Drugs in a Competitive Bodybuilder. Heart, 2005, 91 (7), 888–888. https://doi.org/10.1136/hrt.2004.053843.	Levothyroxine was taken chronically in doping and caused cardiomyopathy which was not lethal.	21
3	Chen, Y.-C.; Fang, J.-T.; Chang, C.-T.; Chou, H.-H. THYROTOXIC PERIODIC PARALYSIS IN A PATIENT ABUSING THYROXINE FOR WEIGHT REDUCTION. Renal Failure, 2001, 23 (1), 139–142.	Levothyroxine was taken as doping and caused thyrotoxic periodic paralysis which was not lethal.	22

	https://doi.org/10.1081/jdi-100001294.		
4	da Silva, J. A.; Almeida, J. T.; Corrêa, B. B.; Narigão, M.; Xavier, M. Acute Psychotic Episode in a Patient with Thyrotoxicosis Factitia. BMJ Case Reports, 2009, 2009, bcr0820080676. https://doi.org/10.1136/bcr.08.2008.0676.	Levothyroxine was taken chronically in case of weight loss and caused psychosis which was not lethal.	23
5	Hartung, B.; Schott, M.; Daldrup, T.; Ritz-Timme, S. Lethal Thyroid Storm after Uncontrolled Intake of Liothyronine in Order to Lose Weight. Int J Legal Med, 2010, 124 (6), 637–640. https://doi.org/10.1007/s00414-010-0423-y.	Liothyronine was taken in acute high dose to commit suicide and led to the occurrence of heart failure. Intoxication was lethal.	24
6	Cantrell, L. Redotex® Revisited: Intentional Overdose with an Illegal Weight Loss Product. The Journal of Emergency Medicine, 2012, 43 (2), e147–e148. https://doi.org/10.1016/j.jemermed.2011.07.023.	Liothyronine was taken in acute high dose to reduce weight and caused tachycardia. Intoxication was not lethal.	25
7	Allen, K. M.; Crawford, V. B.; Conaglen, J. V.; Elston, M. S. Case Report: Clues to the Diagnosis of an Unsuspected Massive Levothyroxine Overdose. CJEM, 2015, 17 (6), 692–698. https://doi.org/10.1017/cem.2014.75.	Levothyroxine was taken in acute high dose to commit suicide and led to the occurrence of seizure. Intoxication was not lethal.	26
8	Bains, A.; Brosseau, A.-J.; Harrison, D. Iatrogenic Thyrotoxicosis Secondary to Compounded Liothyronine. CJHP, 2015, 68 (1). https://doi.org/10.4212/cjhp.v68i1.1426.	Liothyronine was taken chronically due to hypothyroidism and caused iatrogenic STEMI which was not lethal.	27

9	Bains, A.; Brosseau, A.-J.; Harrison, D. Iatrogenic Thyrotoxicosis Secondary to Compounded Liothyronine. CJHP, 2015, 68 (1). https://doi.org/10.4212/cjhp.v68i1.1426.	Liothyronine was taken chronically due to hypothyroidism and caused iatrogenic delirium which was not lethal.	27
10	Rothberger, G. D.; K. Desai, A.; Sharif, S.; A. Chawla, S.; Shirazian, S. The Case ǀ Elevated Lactate and Osmolar Gap after Levothyroxine Overdose. Kidney International, 2015, 88 (2), 419–420. https://doi.org/10.1038/ki.2014.200.	Levothyroxine was taken chronically and in acute high dose to commit suicide and led to the occurrence of delirium. Intoxication was not lethal.	28
11	Kwak, T.; Al Zoubi, M.; Bhavith, A.; Rueda Rios, C.; Kumar, S. Acute Myocarditis in Bodybuilder from Coxsackievirus and Thyrotoxicosis. Journal of Cardiology Cases, 2016, 14 (4), 123–126. https://doi.org/10.1016/j.jccase.2016.06.005.	Unknown substance was taken chronically caused STEMI and was not lethal.	29
12	D’Arcy, R.; McDonnell, M.; Spence, K.; Courtney, C. H. Exogenous T3 Toxicosis Following Consumption of a Contaminated Weight Loss Supplement. Endocrinology, Diabetes & Metabolism Case Reports, 2017, 2017. https://doi.org/10.1530/edm-17-0087.	Unknown substance was taken in acute high dose to reduce weight caused a tachycardia. Intoxication was not lethal	30
13	Xue, J.; Zhang, L.; Qin, Z.; Li, R.; Wang, Y.; Zhu, K.; Li, X.; Gao, X.; Zhang, J. No Obvious Sympathetic Excitation after Massive Levothyroxine Overdose. Medicine, 2018, 97 (23), e10909. https://doi.org/10.1097/md.0000000000010909.	Levothyroxine was taken in acute high dose to commit suicide but patient was clinically stable. Intoxication was not lethal.	31

14	Roomi, S.; Ullah, W.; Iqbal, I.; Ahmad, A.; Saleem, S.; Sattar, Z. Thyrotoxicosis Factitia: A Rare Cause of Junctional Rhythm and Cardiac Arrest. Journal of Community Hospital Internal Medicine Perspectives, 2019, 9 (3), 258–263. https://doi.org/10.1080/20009666.2019.1618668.	Levothyroxine was taken in acute high dose as doping caused thyrotoxicosis. Intoxication was lethal.	32
15	Daher, G.; Hassanieh, I.; Malhotra, N.; Alderson, L. Acute Decompensated Heart Failure Secondary to Exogenous Triiodothyronine Use in a Young Non-Athlete Weightlifter. Cureus, 2019. https://doi.org/10.7759/cureus.5964.	Liothyronine was taken in acute high dose in doping caused heart failure. Intoxication was not lethal.	33
16	Wong, O.; Wong, A.; Greene, S.; Graudins, A. Prolonged Coma Resulting from Massive Levothyroxine Overdose and the Utility of N-Terminal Prohormone Brain Natriuretic Peptide (NT-proBNP). Clinical Toxicology, 2018, 57 (6), 415–417. https://doi.org/10.1080/15563650.2018.1533639.	Levothyroxine was taken in acute high dose to commit suicide and led to the occurrence of thyroid storm. Intoxication was not lethal.	34
17	Warner, B. E.; Woodrow, C. J.; Pal, A. Delayed Diagnosis of T3 Supplementation in a Bodybuilder Presenting with Tachycardia and Features of Sepsis. BMJ Case Rep, 2020, 13 (1), e232867. https://doi.org/10.1136/bcr-2019-232867.	Liothyronine was taken in one large dose caused tachycardia. Intoxication was not lethal.	35
18	Patel, A. J.; Tejera, S.; Klek, S. P.; Rothberger, G. D. Thyrotoxic Periodic Paralysis In A Competitive Bodybuilder With Thyrotoxicosis Factitia. AACE Clinical Case Reports, 2020, 6 (5), e252–e256. https://doi.org/10.4158/accr-2020-0154.	Unknown substance was taken in one large dose caused thyrotoxicosis factitia. Intoxication was not lethal.	36

19	Kiran Kumar, K. C.; Ghimire, N.; Limbu, T.; Khapung, R. Levothyroxine Overdose in a Hypothyroid Patient with Adjustment Disorder: A Case Report. Annals of Medicine and Surgery, 2020, 59, 234–236. https://doi.org/10.1016/j.amsu.2020.09.045.	Levothyroxine was taken in acute high dose to commit suicide and led to the occurrence of tingling sensations in patient's body. Intoxication was not lethal.	37
20	van Bokhorst, Q.; Krul-Poel, Y.; Smit, D.; de Ronde, W. A 29-Year-Old Bodybuilder with Liothyronine-Induced Thyrotoxic Hypokalaemic Periodic Paralysis. European Journal of Case Reports in Internal Medicine, 2021, 8 (3). https://doi.org/10.12890/2021_002362.	Liothyronine was taken in chronic and acute high dose caused thyrotoxic periodic paralysis and was not lethal.	38
21	Bonnar, C. E.; Brazil, J. F.; Okiro, J. O.; Giblin, L.; Smyth, Y.; O'Shea, P. M.; Finucane, F. M. Making Weight: Acute Muscle Weakness and Hypokalaemia Exacerbated by Thyrotoxicosis Factitia in a Bodybuilder. Endocrinology, Diabetes & Metabolism Case Reports, 2021, 2021. https://doi.org/10.1530/edm-21-0060.	Levothyroxine was taken in acute high dose caused thyrotoxic periodic paralysis. Intoxication was not lethal.	39
22	Du, F.; Liu, S.-W.; Yang, H.; Duan, R.-X.; Ren, W.-X. Thyrotoxicosis after a Massive Levothyroxine Ingestion: A Case Report. WJCC, 2022, 10 (11), 3624–3629. https://doi.org/10.12998/wjcc.v10.i11.3624.	Levothyroxine was taken in acute high dose to commit suicide led to atrial fibrillation. Intoxication was not lethal.	40
23	Du, F.; Liu, S.-W.; Yang, H.; Duan, R.-X.; Ren, W.-X. Thyrotoxicosis after a Massive Levothyroxine Ingestion: A Case Report. WJCC, 2022, 10 (11), 3624–3629. https://doi.org/10.12998/wjcc.v10.i11.3624.	Levothyroxine was taken in acute high dose to commit suicide led to atrial fibrillation. Intoxication was not lethal.	41

24	Gill, A. S.; Rai, H. K.; Karunakaran, A.; Chaudhuri, A. Suicide Attempt With Levothyroxine Overdose. Cureus, 2023. https://doi.org/10.7759/cureus.36172.	Levothyroxine was taken in acute high dose to commit suicide led to atrial fibrillation. Intoxication was not lethal.	42
25	Momoh, R.; Hassan, A. A Case Report of an Acute Severe Tachyarrhythmia Presentation With Underlying Cardiomyopathy in a Patient With Anabolic Androgenic Steroid and Thyroxine Misuse. Cureus, 2024. https://doi.org/10.7759/cureus.62806.	Levothyroxine was taken in acute high dose caused tachycardia with severe palpitation and sedation. Intoxication was not lethal.	43
26	Sowmya Durga; Subhasri Guna; Aishwar Dixit; Shruthi Katam; Odoch, A. K.; Jeyasundar Dhevanbu; Hassan, M.; Sowah, A. O.; Tochukwu Anthony Akwue; Wondimagegn Tibebu Tilahun. Acute MI of Young Age: Unwarranted Dosing of Steroidal and Nutritional Supplementations. Unpublished 2024. https://doi.org/10.13140/RG.2.2.29205.74728.	Liothyronine was taken in acute high dose caused STEMI. Intoxication was not lethal.	44
27	Theocharidou, C.-C.; Pavlidou, M.; Endiaroglou, M.; Dimaki, A.; Ampatzidou, F. Coma as the Sole Initial Manifestation of Levothyroxine Intoxication: A Case Report. The Journal of Emergency Medicine, 2025, 75, 319–322. https://doi.org/10.1016/j.jemermed.2025.03.005.	Levothyroxine was taken in acute high dose to commit suicide resulted in coma which was not lethal.	44
28	He, Z. H.; Li, Y.; Trivedi, N.; Gill, S.; Hennessey, J. V. Thyrotoxicosis after Massive Triiodothyronine (LT3) Overdose: A Coast-to-Coast Case Series and Review. DIC, 2020, 9, 1–5. https://doi.org/10.7573/dic.2019-8-4.	Liothyronine was taken in high dose due to iatrogenic error and caused tachycardia. Intoxication was not lethal.	45

29	He, Z. H.; Li, Y.; Trivedi, N.; Gill, S.; Hennessey, J. V. Thyrotoxicosis after Massive Triiodothyronine (LT3) Overdose: A Coast-to-Coast Case Series and Review. DIC, 2020, 9, 1–5. https://doi.org/10.7573/dic.2019-8-4.	Liothyronine was taken in acute high dose due to iatrogenic error and caused nausea, vomiting and general malaise. Intoxication was not lethal.	45
30	He, Z. H.; Li, Y.; Trivedi, N.; Gill, S.; Hennessey, J. V. Thyrotoxicosis after Massive Triiodothyronine (LT3) Overdose: A Coast-to-Coast Case Series and Review. DIC, 2020, 9, 1–5. https://doi.org/10.7573/dic.2019-8-4.	Compounded liothyronine/ levothyroxine was taken in acute high dose due to iatrogenic error and caused tachycardia. Intoxication was not lethal.	45
31	Vorasart, P.; Sriphrapradang, C. Factitious Thyrotoxicosis: How to Find It. Diagnosis, 2019, 7 (2), 141–145. https://doi.org/10.1515/dx-2019-0015.	Levothyroxine was taken chronically to reduce symptoms. It resulted in facetious thyrotoxicosis which was not lethal.	46
32	Nanjappa, H.; Rodrigues, A. J. Atrial Fibrillation Secondary to Levothyroxine Overdose with Underlying Secondary Infection. Journal of The Association of Physicians of India, 2024, 72 (8), 96–98. https://doi.org/10.59556/japi.72.0602.	Levothyroxine taken chronically caused atrial fibrillation. Intoxication was not lethal.	47
33	Li, R.; Xu, Y.-W.; Xue, Y.; Wu, X.-Z. Plasmapheresis in the Treatment of Multi-Drug Intoxication Involving Levothyroxine Sodium and Calcium Channel Blockers: A Case Report. Ann Palliat Med, 2020, 9 (5), 69–69. https://doi.org/10.21037/apm-20-190.	Levothyroxine was taken in acute high dose to commit suicide and led to the occurrence of the dyspnea, vomiting and low blood pressure which was not lethal.	48
34	de Luis, D. A.; Dueñas, A.; Martin, J.; Abad, L.; Cuellar, L.; Aller, R. Light Symptoms Following a High-Dose Intentional <I>L</I>-Thyroxine Ingestion Treated with Cholestyramine. Horm Res	Levothyroxine was taken in acute high dose to commit suicide, and led to the occurrence of the distal tremor and diaphoresis which was not lethal.	49

	Paediatr, 2002, 57 (1–2), 61–63. https://doi.org/10.1159/000057950.		

Supplementary Table S3. Summary of other substances used concurrently with thyroid hormones as doping in individual clinical case reports.

Case ID	Substances
2	unknown
14	testosterone, oxandrolone, methenolone
15	clenbuterol, testosterone enanthate, trenbolone,
17	testosterone
18	trenbolone, testosterone enanthate, boldenone undecylenate, stanozolol
20	testosterone enanthate, masteron enanthate, methandienone, human growth hormone
21	trenbolone, stanozolol, oxandrolone, mesterolone, fluoxymesterone and drostanolone, growth hormone, clenbuterol, letrozole
25	testosterone
26	stanozolol, testosterone

Supplementary Table S4. Summary of medicines used concurrently with thyroid hormones as doping in individual clinical case reports.

Case ID	Substances
11	unknown
17	propranolol, propylthiouracil
18	furosemide
25	valsartan, bisoprolol, dapagliflozin

Supplementary Table S5. Summary of medicines used concurrently with thyroid hormones not used as doping in individual clinical case reports.

Case Report	Reason for abuse	Substances
7	suicide	arotinolol
9	suicide	citalopram
12	suicide	unknown
13	suicide	eletriptan, diazepam
16	suicide	propranolol, sertraline
19	suicide	clonazepam, zolpidem
22	suicide	clonazepam, zolpidem
23	suicide	hydrocortisone, testosterone, bupropion, enalapril
27	suicide	diltiazem, amlodypin
29	to reduce symptoms	amlodipine, pantoprazole
30	to reduce symptoms	perindopril, metformin, acetylsalicylic acid
31	to reduce symptoms	radioactive iodine treatment
33	weight loss	atropine, d-norpseudoephedrine, diazepam
34	weight loss	sibutramine

Supplementary Table S6. The number of thyroid hormone poisoning cases depending on how they were used.

Diagnosis	Chronic use	Chronic use and acute high dose	One acute high dose
Atrial fibrillation	0	2	0
Cardiogenic shock	0	0	1
Cardiomyopathy	1	0	0
Coma	0	1	1
Delirium	1	1	0
Dyspnea	0	0	1
Heart failure	0	0	2
No symptoms of thyroid toxicity	0	2	0

Psychosis	1	0	0
Seizure	0	2	0
ST-elevation myocardial infarction (STEMI)	2	0	1
Tachycardia	1	4	4
Thyrotoxic periodic paralysis	0	1	2
Thyrotoxicosis	1	0	0
Tremor	0	1	0
Total	7	14	12

Note: Percentage values not presented because of very small subclass sizes

Supplementary Table S7. The number of thyroid hormone poisoning cases by drug type.

Diagnosis	compounded liothyronine/levothyroxine	levothyroxine	liothyronine
Atrial fibrillation	0	2	0
Cardiogenic shock	0	1	0
Cardiomyopathy	0	1	0
Coma	0	2	0
Delirium	0	1	1
Dyspnea	0	1	0
Heart failure	0	0	2
No symptoms of thyroid toxicity	0	2	0
Psychosis	0	1	0
Seizure	0	2	0
ST-elevation myocardial infarction (STEMI)	0	0	2
Tachycardia	1	3	4
Thyrotoxic periodic paralysis	0	2	1
Thyrotoxicosis	0	1	0
Tremor	0	1	0
Total	1	20	10

Note: Percentage values not presented because of very small subclass sizes
